# Induction of tumour blood flow stasis and necrosis: a new function for epinephrine similar to that of combretastatin A-4 derivative AVE8062 (AC7700)

**DOI:** 10.1038/sj.bjc.6601582

**Published:** 2004-01-20

**Authors:** K Hori, S Saito

**Affiliations:** 1 Department of Vascular Biology, Division of Cancer Control, Institute of Development, Aging and Cancer, Tohoku University, 4-1 Seiryomachi, Aoba-ku, Sendai 980-8575, Japan

**Keywords:** epinephrine, combretastatin A-4, tumour blood flow, tumour vessel, necrosis

## Abstract

AVE8062, a derivative of combretastatin A-4, has a strong stanching effect on tumour blood flow (TBF), which leads to complete blockage of nutrient supply to solid tumours and their necrosis. Previously, we reported that TBF stasis is due to increased arteriolar resistance caused by AVE8062 and a lasting decrease in perfusion pressure in tumour-feeding vessels. Here, we measured changes in TBF in rat solid tumour LY80 during continuous administration of AVE8062-like epinephrine or four catecholamines that are unlike AVE8062 (norepinephrine, dopamine, methoxamine, and metaraminol) to the region of increased vascular resistance. Venous administration of 0.3 mg ml^−1^ epinephrine caused TBF to fall immediately to near zero, where it remained throughout the administration period. With a 30-min drug administration, TBF began to recover immediately when drug administration halted. With a 60-min epinephrine administration, TBF recovered somewhat, but not to the previous level. With drug administration of 120 min, TBF did not recover during the subsequent 8 h. Likewise, 0.1 mg ml^−1^ epinephrine produced irreversible occlusion after 120 min of administration. In contrast, 120 min of administration of the four other catecholamines resulted in no occlusion. Only the group given 0.3 mg ml^−1^ epinephrine (not that given methoxamine) showed significantly greater necrosis than the control. We conclude that, for epinephrine to cause irreversible occlusion of these vessels, a marked decrease in perfusion pressure in tumour-feeding blood vessels is necessary and should be maintained for 2 h. This conclusion is consistent with the previously demonstrated mechanism of irreversible arteriole occlusion caused by AVE8062. AVE8062 and epinephrine appear to have the same mechanism of action regarding induction of tumour blood flow stasis.

AVE8062 (previous code name AC7700), a derivative of combretastatin A-4, is reported to cause powerful stasis of tumour blood flow (TBF) (Hori *et al*, 1999), thereby preventing nutrient supply to the cancer and ultimately leading to necrosis of solid tumours (Hori *et al*, 1999; Nihei *et al*, 1999a, 1999b). Such effects are known for subcutaneously transplanted mouse and rat tumours, autochthonous primary tumours induced by chemical carcinogens (Hori *et al*, 2001), tumours growing within internal organs (Hori *et al*, 2002; Ohno *et al*, 2002), lymph node metastases, small foci cancers, and most solid tumours (Hori *et al*, 2002; Hori, 2003).

Previously, we showed by means of analysis of microcirculation and intravital microscopic observations that the vascular occlusion is not due to a direct effect of AVE8062 on tumour vessels, but rather is likely due to an indirect effect on host arterioles (Hori and Saito, 2003). Thus, AVE8062 causes a lasting increase in vascular resistance of host arterioles, and this effect produces a marked and persistent fall in the perfusion pressure of the downstream vessels feeding the tumour. As a consequence, the lumens of many tumour vessels become constricted or disappear entirely. Many red blood cells then form aggregates in the lumens of drainage vessels, and after 2–3 h haemolysis of the red blood cells and subsequent thrombosis occur. This is thought to be the irreversible process of tumour vessel occlusion caused by AVE8062 (Hori and Saito, 2003).

If this microcirculatory mechanism is a general one, then drugs other than AVE8062 that produce persistent blockage of blood flow in tumour-feeding vessels should show irreversible TBF stanching, similar to that caused by AVE8062. To test this hypothesis, we measured the effect of five catecholamines: epinephrine, norepinephrine, dopamine, and metaraminol on tumour blood flow. Arteries can be clasiffied according to Strahler’s method (1957): capillaries branching off from terminal arterioles are assigned index number 1, terminal arterioles are defined as a2; arterioles are then defined as a3, a4, a5 and so on, as one proceeds from the periphery (Hori *et al*, 1990). Both AVE8062 and epinephrine raise the vascular resistance of host arterioles a3, a4, and a5, and halt the blood flow in the downstream vessels that feed the tumour (a2 vessels modified because of the influence of the tumour) (Hori *et al*, 1993; Hori and Saito, 2003). In contrast, methoxamine (Hori *et al*, 1993), norepinephrine, dopamine, and metaraminol have less effect on vascular resistance in a3, a4, and a5 vessels, and do not block blood flow in tumour-feeding vessels (data not shown).

The purpose of the present research was to demonstrate that, when blood flow of tumour-feeding vessels is blocked for a fixed time period by epinephrine, irreversible stanching of blood flow to the tumour and widespread necrosis occur, similar to the effects of AVE8062, and also that, when hypertensive catecholamines, which cannot block the blood flow of tumour-feeding vessels, such as norepinephrine, dopamine, methoxamine, or metaraminol are used, occlusion of tumour vessels does not occur.

## MATERIALS AND METHODS

### Rats and tumours

Male Donryu rats (Crj-Donryu; Nippon Charles-River, Yokohama, Japan), 8–10 weeks old and with an average weight of 250–300 g, were used for all experiments. Rats were bred and maintained in a ventilated, temperature-controlled (24±1°C), specific pathogen-free environment, on a bed of wood shavings, with food and water freely available and a 12-h light–dark cycle.

The tumour cell line LY80, which is a variant of Yoshida sarcoma, was used. In our laboratory, this cell line is maintained by successive i.p. transplantation. LY80 cells growing in ascites of a donor rat were collected, suspended in phosphate-buffered saline, and adjusted to a concentration of 2 × 10^6^ cells per 0.1 ml. Recipient rats were anaesthetised with diethyl ether (Wako Pure Chemical Industries, Ltd, Osaka, Japan). Tumour cells (2 × 10^6^ cells) were implanted into the s.c. tissue of the back of the rats.

Experiments were performed with the animals anaesthetised in a temperature-controlled (24±1°C) box fitted with a suction duct. All experimental protocols were reviewed by the Committee on the Ethics of Animal Experiments in our institute, and were carried out in accordance with Guidelines for Animal Experiments issued by Tohoku University School of Medicine and The Law (No. 105) and Notification (No. 6) issued by the Japanese Government. The ethical guidelines that were followed meet the standards required by the UKCCCR (Workman *et al*, 1998) guidelines.

### Drugs

Epinephrine at 0.3 and 0.1 mg ml^−1^ (Bosmin; Daiichi Pharmaceutical Co., Ltd, Tokyo, Japan), 0.3 mg ml^−1^ norepinephrine (Noradrenaline; Sankyo Co., Ltd, Tokyo, Japan), 5 mg ml^−1^ dopamine (Inovan; Kyowa Hakko Kogyo Co., Tokyo, Japan), 2 mg ml^−1^ methoxamine HCl (Mexan; Nippon Shinyaku Co., Ltd, Kyoto, Japan), and 1 mg ml^−1^ metaraminol bitartrate (Araminon; Banyu Pharmaceutical Co., Ltd, Tokyo, Japan) were used as vasopressors. A solution of 0.9% NaCl (Otsuka Pharmaceutical Co., Ltd, Tokyo, Japan) was used as a control. Each drug was used at the concentration at which systemic blood pressure increased to 150 mmHg within 2 min after the start of i.v. drug administration at a rate of 0.015 ml min^−1^.

Pentobarbital sodium salt (Tokyo Kasei Kogyo Co., Ltd, Tokyo, Japan) and enflurane (Ethrane; Abbott Laboratories, North Chicago, IL, USA) were given simultaneously for anaesthesia. The pentobarbital powder was dissolved in distilled water to give a concentration of 50 mg ml^−1^, and was administered i.m. 10 min before the experiment at a dose of 30 mg kg^−1^. Supplemental doses (15 mg kg^−1^ i.m.) were given at 90-min intervals to maintain immobilisation. The enflurane concentration was kept at 1% (v v^−1^) in the inhaled carrier gas, which was provided at a rate of 1 l min^−1^ by means of an anaesthetic apparatus for small laboratory animals. We certify that this anaesthetic technique did not change blood pressure or TBF significantly throughout the experiment (Hori *et al*, 2002).

### Measurement of TBF

TBF was measured by use of the hydrogen-clearance method (Hori *et al*, 1991, 2002) that was originally developed by Aukland *et al* (1964). The blood flow measured by this method is not the total blood flow (Gullino and Grantham, 1961); rather, it is the local blood flow (Peterson, 1979). In brief, after saturation of the tissue with hydrogen by means of inhalation of 9% hydrogen gas in air (at 1 l min^−1^), the blood flow value (in ml min^−1^ per 100 g tissue) in a small region was calculated from the half-life of the clearance curve obtained. A tissue blood flow metre with two separate amplifiers (PHG-201; Unique Medical Co., Tokyo, Japan) was used. Two 80-*ì*m-diameter hydrogen electrodes (UHE-201C; Unique Medical) and two rod-type Ag/AgCl reference electrodes (TT-98012; Unique Medical) were usually used for each rat. The reference electrodes were inserted between the skin and the musculature in the caudal region.

TBF was measured 8–10 days after tumour implantation. For this measurement, electrodes were inserted to a depth of 2 mm from the tumour surface. The drug was continuously infused for 120 min at a rate of 0.015 ml min^−1^ by using a microinfusion pump (Compact Syringe Pump; Harvard Apparatus Co., Inc., Millis, MA, USA). TBF was measured every 10 min for 30 min, then every 30 min for 1.5 h, and thereafter every hour for 6 h. Throughout the experiment, rats were placed prone on a heated stage at 34°C and were kept in the same position. Rectal temperature, monitored with a thermistor for small animals (PTC-201; Unique Medical), was 33.5–35.5°C and the condition was maintained.

### Measurement of mean arterial blood pressure

Mean arterial blood pressure (MABP) was monitored in all rats in which TBF was measured. MABP was measured via a catheter (PE-50; Becton Dickinson and Company, Sparks, MD, USA) inserted into the right femoral artery. Pressure in the catheter was recorded with a pressure transducer (TNF-R; Spectramed Medical Products, Singapore), the output of which was fed into an amplifier (6M82; NEC-Sanei Co., Tokyo, Japan) adapted for such measurement.

### Histological examination

LY80 tumour-bearing rats were divided into three groups: I, a group receiving 0.3 mg ml^−1^ epinephrine solution (four rats); II, a group receiving 2 mg ml^−1^ methoxamine solution (four rats); and III, a group receiving 0.9% NaCl solution (four rats). Each solution was continuously infused for 120 min into the tail vein at a rate of 0.015 ml min^−1^ by using a microinfusion pump (Compact Syringe Pump; Harvard Apparatus). Rats in all groups were killed with ether 48 h after treatment. Each tumour was resected for routine histology, fixed in 15% formalin, processed, and embedded in paraffin. Sections of each tumour were cut 4 *μ*m thick and were stained with haematoxylin and eosin. The area and distribution ratio of the necrotic regions were calculated by using an area analyser (WT-4400SE, WACOM Co., Saitama, Japan).

### Statistics

All results are expressed as means±s.d. The statistical significance for the change in TBF at each time point induced by each vasoactive drug compared with that induced by 0.9% NaCl solution was evaluated via the unpaired two-group *t*-test. The occurrence of necrosis induced by the administration of epinephrine or methoxamine was also compared by using the unpaired two-group *t*-test. *P*-values of 0.05 or lower were considered statistically significant.

## RESULTS

### Effect of continuous i.v. administration of epinephrine on TBF and MABP

**Figure 1:**
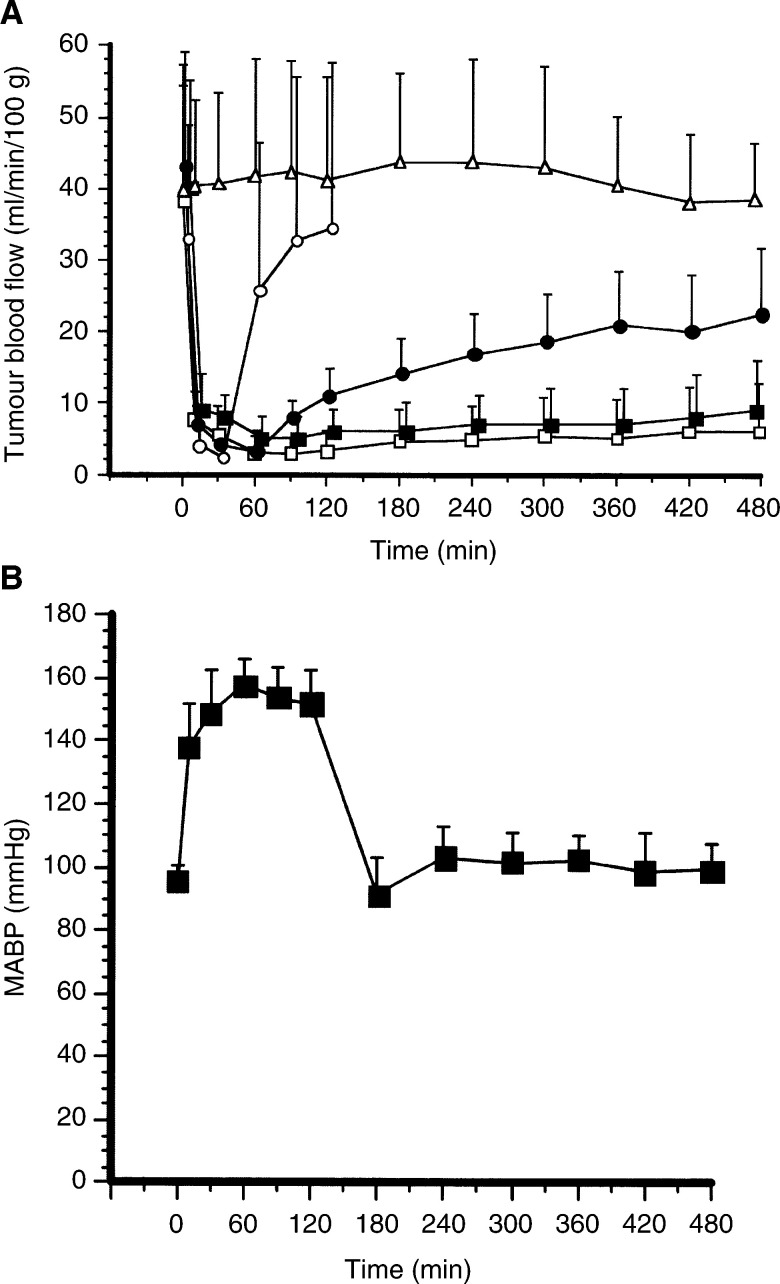
Changes in TBF and systemic pressure (MABP) caused by epinephrine. (**A**) Changes in TBF after continuous administration of epinephrine at a rate of 0.015 ml min^−1^. ○, 30-min administration of 0.3 mg ml^−1^ epinephrine (*n*=10); •, 60-min administration of 0.3 mg ml^−1^ epinephrine (*n*=10); □, 120-min administration of 0.3 mg ml^−1^ epinephrine (*n*=14); ▪, 120-min administration of 0.1 mg ml^−1^ epinephrine (*n*=10); △, 0.9% NaCl solution (*n*=12). Continuous administration began at time 0. (**B**) Changes in systemic pressure caused by 120-min continuous administration of 0.3 mg ml^−1^ epinephrine.

The changes in TBF during and after continuous i.v. administration of 0.3 mg ml^−1^ epinephrine for 30, 60, and 120 min, and of 0.1 mg ml^−1^ epinephrine for 120 min, compared with TBF change during and after 0.9% NaCl administration are shown in Figure 1A

. TBF did not change at all by 0.9% NaCl. In contrast, TBF decreased immediately and significantly (*P*<0.01) after the start of i.v. administration of 0.3 mg ml^−1^ epinephrine, and this level was maintained during the continuous administration of the drug. In the case of drug administration for 30 min, the decreased TBF started to recover immediately after the end of drug administration and returned to the initial level approximately 90–120 min later. With 60 min of drug administration, TBF began to recover gradually beginning 60 min after the end of drug administration. However, TBF did not return to the initial level, even 8 h later. With 120 min of drug administration, the TBF did not recover at all throughout the experimental period, to 6 h after the end of the drug administration. Irreversible stasis of TBF was also observed at the concentration of 0.1 mg ml^−1^ epinephrine, with continuous administration for 120 min.

The change in MABP during and after continuous i.v. administration of 0.3 mg ml^−1^ epinephrine for 120 min (seven rats) is shown in Figure 1B. MABP was maintained at 150.1±7.5 mmHg during the administration of the drug. However, the increased MABP decreased immediately after the end of drug administration.

### Effect on TBF of continuous i.v. administration of four catecholamines for 120 min

Figure 2

**Figure 2:**
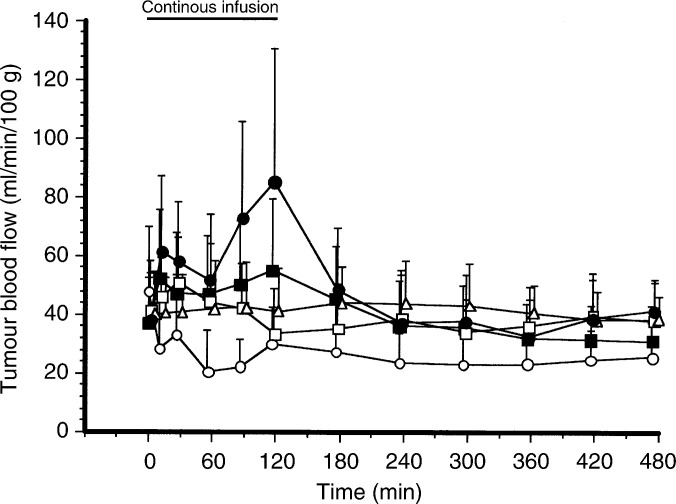
Changes in TBF after 120 min of continuous administration of various catecholamines: ○, 0.3 mg ml^−1^ norepinephrine (*n*=10); •, 5 mg ml^−1^ dopamine (*n*=8); □, 2 mg ml^−1^ methoxamine (*n*=11); ▪, 1 mg ml^−1^ metaraminol (*n*=10); △, 0.9% NaCl solution (*n*=12, identical with Figure 1).

indicates TBF changes during and after 120-min continuous i.v. administration of 0.3 mg ml^−1^ norepinephrine, 5 mg ml^−1^ dopamine, 2 mg ml^−1^ methoxamine, 1 mg ml^−1^ metaraminol, or 0.9% NaCl solution. TBF in the methoxamine-treated group significantly increased for 30 min after the start of drug administration compared with TBF in the 0.9% NaCl group (*P*<0.01). TBF in the metaraminol-treated and dopamine-treated groups significantly increased during drug administration, compared with TBF in the 0.9% NaCl group (*P*<0.01). In contrast, TBF in the norepinephrine-treated group significantly decreased by approximately 50% throughout the experimental period of 8 h compared with TBF in the 0.9% NaCl group (*P*<0.01).

### Histological examination

Figure 3

**Figure 3:**
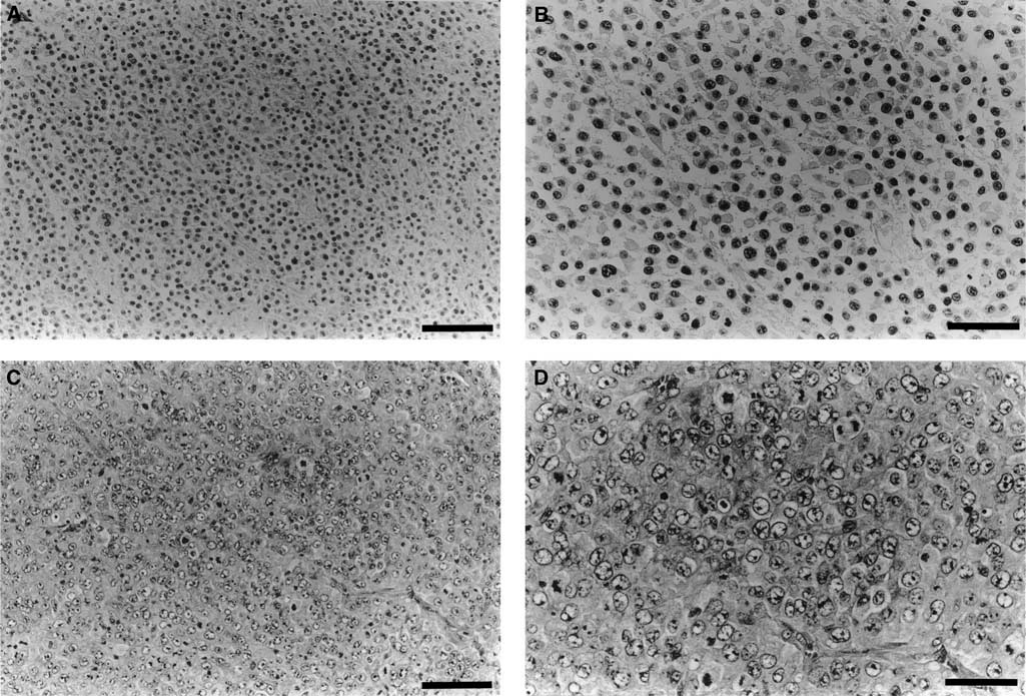
Effects of continuous administration of epinephrine or methoxamine on tumour tissue. (**A**, **B**) show the effects of epinephrine; (**C**, **D**) show those of methoxamine. (**A**, **C**) show 200-fold magnifications; bars, 100 *μ*m. (**B**, **D**) show 400-fold magnifications; bars, 50 *μ*m.

shows the typical histology of an LY80 tumour 48 h after continuous i.v. administration for 120 min of 0.3 mg ml^−1^ epinephrine or 2 mg ml^−1^ methoxamine, at a rate of 0.015 ml min^−1^. Extensive necrosis was found in the epinephrine-treated group (Figure 3A and B), but no large necrotic area was seen in the methoxamine-treated group (Figure 3 C and D). The percentage of necrosis in the maximum tumour section is shown in Figure 4

**Figure 4:**
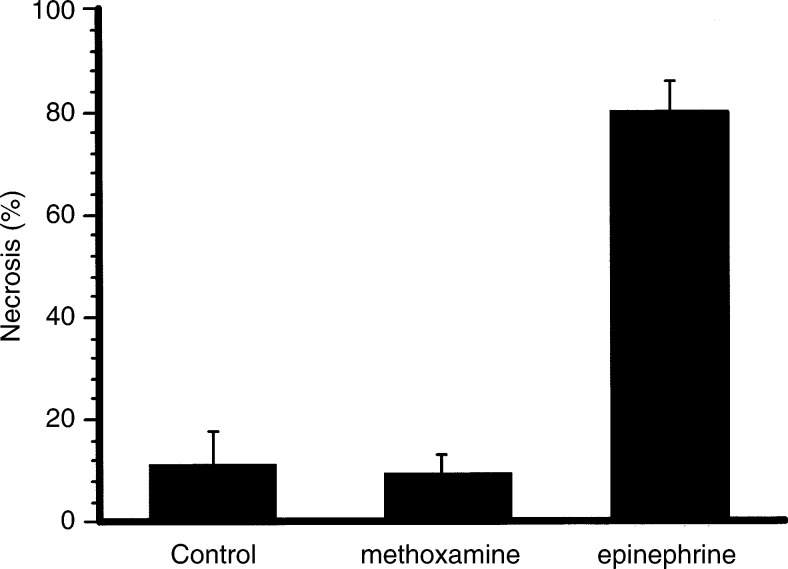
Percentage of necrosis 48 h after continuous administration of epinephrine or methoxamine. A significant difference in necrosis was not found between the methoxamine and 0.9% NaCl-treated groups, but there was a significant increase in necrosis in the epinephrine-treated grouped compared with the methoxamine-treated and 0.9% NaCl-treated groups.

. The percentages of necrosis after treatment with epinephrine (*n*=4), methoxamine (*n*=4), or 0.9% NaCl solution (*n*=4) were 79.7±5.9% (tumour area, 1.43±0.37 cm^2^), 8.5±4.5% (tumour area, 1.47±0.20 cm^2^), and 9.6±8.1% (tumour area, 1.50±0.24 cm^2^), respectively. Highly significant necrosis was thus observed in the epinephrine-treated group (epinephrine *vs* methoxamine and epinephrine *vs* 0.9% NaCl, both at *P*<0.001). The difference between the methoxamine-treated group and the control group was not significant, however. On the other hand, epinephrine showed no histological effects on normal tissues under the condition described above (data not shown).

## DISCUSSION

In the present study, we showed that, when a marked decrease in perfusion pressure in tumour-feeding vessels is brought about with the use of epinephrine, vascular blood flow stanching becomes irreversible after 2 h, which leads to widespread necrosis within the tumour. Since cultures of LY80 cells grown in a medium containing 0.03–0.3 mg ml^−1^ epinephrine for more than 2 h did not show any decrease in cancer cell viability (data not shown), we conclude that the epinephrine-induced tumour necrosis was clearly a consequence of the effect of epinephrine on tumour microcirculation.

Clinically, epinephrine is currently used during resuscitation following cardiac arrest or for treatment of anaphylaxis. In the field of cancer research, epinephrine is known to reduce TBF (Li *et al*, 1990; Hori *et al*, 1993). Moreover, in pharmacoangiography, which exploits the different responses of tumour tissue and normal tissue, attempts at improving the diagnostic image by using epinephrine had been reported long ago (Abrams, 1964). The ability of epinephrine to induce selective tumour necrosis by means of continuous administration has not previously been reported, and is thought to be the principal new finding of the present study.

This conclusion was made deductively on the basis of analysis of haemodynamic mechanisms leading to the necrosis of solid tumours by the derivative of combretastatin A-4, that is, AVE8062 (Hori and Saito, 2003). AVE8062 also induces an increase in systemic blood pressure (Hori *et al*, 1999), similar to that produced by epinephrine, but the hypertension itself does not cause the stanching of TBF. In methoxamine, dopamine, and metaraminol, there was no stanching effect even after 120 min of continuous drug administration and did not induce necrosis of tumour tissue. Whether stasis of TBF occurs depends on which arterioles show increases in vascular resistance. The present study achieved the clarification of this point. However, it is not yet clear from the present studies as to why methoxamine, dopamine, and metaraminol have a lesser effect on the a3, a4, and a5 arterioles.

Although the sites at which AVE8062 and epinephrine facilitate the increase in vascular resistance are similar, the decisive difference between these two agents is the duration of their effects. The effects of AVE8062, after one-shot administration, persisted for 2–3 h (Hori *et al*, 1999), whereas the effects of epinephrine disappeared immediately when administration was halted. We therefore hypothesised that, if the duration of epinephrine-induced increased vascular resistance would approach that of AVE8062, epinephrine should also bring about tumour necrosis in a manner similar to that of AVE8062. We consequently investigated the effects of 30, 60, and 120 min of continuous administration of epinephrine on changes in blood flow to tumour tissue. We found that TBF began to recover immediately after cessation of 30 min of epinephrine administration, whereas, after a 60-min administration, the TBF showed some recovery, but not to its original level. After 120 min of epinephrine administration, however, TBF did not recover and the stasis was irreversible. Moreover, similar to AVE8062, epinephrine induced extensive intratumour necrosis. Noteworthy is the fact that, although a dosage of 0.3 mg ml^−1^ epinephrine delivered during 60 min did not produce irreversible occlusion of tumour blood vessels, a lower dosage, 0.1 mg ml^−1^, delivered for 120 min did produce irreversible effects. This fact demonstrates that the total amount of epinephrine administered is not important, but the duration of administration is.

With regard to tumour growth inhibition, epinephrine was notably weaker than AVE8062 (data not shown). Epinephrine had antitumour effects related solely to its occlusive effects, whereas AVE8062 not only occluded tumour blood vessels but was also thought to inhibit tubulin polymerisation in tumour cells. This fact suggests that the tumoricidal effects of epinephrine could be significantly enhanced by administration of epinephrine together with appropriate anticancer drugs.

As for the mechanism by which AVE8062 constricts host arterioles, the present research does not provide definite answers. Nevertheless, because the effects of AVE8062 on microcirculation are extremely similar to those of epinephrine, it is likely that both agents affect vascular smooth muscle tissue. Thus, a molecular-level study will be necessary to determine whether a specific receptor exists for AVE8062 or whether the effects on vascular resistance brought about by AVE8062 involve the epinephrine-activated alpha receptor.

From the present results concerning these catecholamines, we can conclude that the conditions necessary for bringing about irreversible occlusion of TBF are as follows: (i) marked reduction of perfusion pressure of vessels feeding the tumour, and (ii) maintenance of hemodynamic stasis for at least 2 h. AVE8062 and epinephrine appear to have the same mechanism of action regarding induction of tumour blood flow stasis.
